# Photoautotrophic production of polyhydroxyalkanoates in a synthetic mixed culture of *Synechococcus elongatus cscB* and *Pseudomonas putida cscAB*

**DOI:** 10.1186/s13068-017-0875-0

**Published:** 2017-07-19

**Authors:** Hannes Löwe, Karina Hobmeier, Manuel Moos, Andreas Kremling, Katharina Pflüger-Grau

**Affiliations:** Fachgebiet für Systembiotechnologie, Technische Universität München, Boltzmannstr 15, 85748 Garching, Germany

**Keywords:** Carbon neutral bioplastics, Polyhydroxyalkanoates (PHA), Synthetic co-culture, *Pseudomonas putida cscAB*, *Synechococcus elongatus cscB*, Cyanobacteria, CO_2_ fixation

## Abstract

**Background:**

One of the major challenges for the present and future generations is to find suitable substitutes for the fossil resources we rely on today. Cyanobacterial carbohydrates have been discussed as an emerging renewable feedstock in industrial biotechnology for the production of fuels and chemicals, showing promising production rates when compared to crop-based feedstock. However, intrinsic capacities of cyanobacteria to produce biotechnological compounds are limited and yields are low.

**Results:**

Here, we present an approach to circumvent these problems by employing a synthetic bacterial co-culture for the carbon-neutral production of polyhydroxyalkanoates (PHAs) from CO_2_. The co-culture consists of two *bio*-*modules*: *Bio*-*module I*, in which the cyanobacterial strain *Synechococcus elongatus cscB* fixes CO_2_, converts it to sucrose, and exports it into the culture supernatant; and *bio*-*module II*, where this sugar serves as C-source for *Pseudomonas putida cscAB* and is converted to PHAs that are accumulated in the cytoplasm. By applying a nitrogen-limited process, we achieved a maximal PHA production rate of 23.8 mg/(L day) and a maximal titer of 156 mg/L. We will discuss the present shortcomings of the process and show the potential for future improvement.

**Conclusions:**

These results demonstrate the feasibility of mixed cultures of *S. elongatus cscB* and *P. putida cscAB* for PHA production, making room for the cornucopia of possible products that are described for *P. putida*. The construction of more efficient sucrose-utilizing *P. putida* phenotypes and the optimization of process conditions will increase yields and productivities and eventually close the gap in the contemporary process. In the long term, the co-culture may serve as a platform process, in which *P. putida* is used as a chassis for the implementation of synthetic metabolic pathways for biotechnological production of value-added products.

**Electronic supplementary material:**

The online version of this article (doi:10.1186/s13068-017-0875-0) contains supplementary material, which is available to authorized users.

## Background

For a long time, natural polymers like wood or wool have been used by humans to craft weapons and tools or to protect against the cold. This enhanced our ability to survive and allowed us to build cultures and to live in places that are hostile to our biology. From the nineteenth century, with the invention of modern polymer chemistry, many of these natural materials were complemented and/or replaced by modern plastics [1]. Plastics found application in all areas of our daily life. In 2015, the global plastics material production was estimated to reach 250 million tons per year [2] and most of the plastics produced were derived from petroleum. Because of the inevitable finiteness of fossil resources and the massive pollution caused by plastic wastes [3], contemporary research in this field is directed towards the exploration of renewable and biologically degradable sources of plastics. Advanced natural polymers like polyhydroxyalkanoates (PHA) that show thermoplastic, polypropylene-like properties, could be a valuable substitute for some applications. Under natural conditions, PHA, a linear polymer of 3-hydroxy fatty acids, serves as both energy and carbon storage in certain bacteria, among them *Pseudomonas putida*. In industrial production of PHA, substrate price is a key factor [4] as bio-based plastics have to compete with those made from fossil resources. Therefore, efforts are directed towards alternative feedstocks to reduce substrate costs in the overall process.

A newly discussed, potentially cheap source of substrates are carbohydrates produced by microalgae and cyanobacteria, for example, starch production by eukaryotic algae or sucrose production by recombinant cyanobacteria [5–8]. Compared to conventional crops, microalgae and cyanobacteria have the potential to reach higher areal yields and, additionally, their products do not interfere with the food markets. Further benefits include the ability to use salty or brackish water and bioreactors can be placed on non-arable land. The genetically engineered cyanobacterial strain *Synechococcus elongatus cscB* has recently been shown to export sucrose on a level comparable to sugar cane [6]. This was achieved by the introduction of only one heterologous gene encoding the sucrose permease CscB from *Escherichia coli* ATCC 700927. Under salt stress, *S. elongatus cscB* accumulates remarkable amounts of sucrose as a compatible solute, which are released into the surrounding medium by the activity of the heterologous permease CscB [6]. However, when these sugars are produced on a large scale, limitations will arise: These include the risk of contamination, as a carbon source is provided that can be used by heterotrophs, and economic aspects, e.g., the cost of sugar recovery from the fermentation broth [9, 10]. A recent approach to circumvent these problems is to convert the cyanobacterial feedstock directly into value-added products in a multispecies microbial factory in a so-called “one-pot” reaction [11]. By co-inoculation of both strains, the sugar-producing strain together with the product accumulating strain, several barriers can be overcome. Thus, the costs for sugar recovery from the cyanobacterial fermentation broth as well as the potential loss due to contamination are saved. This contributes to making the overall process economically more competitive. Furthermore, there are cases in which a positive effect of synthetic consortia on the productivity have been described [12, 13]. Along that line, two studies have been published recently that aimed to produce polyhydroxybuturate (PHB) in a mixed culture of *S. elongatus cscB* with *Escherichia coli* or *Azotobacter vinelandii,* respectively [9, 14]. Even though the final titers reached were quite low (around 1 mg/L), which might be a result of slow growth rates and suboptimal media composition, this shows the feasibility of the general approach and leaves room for improvement.

**Fig. 1:**
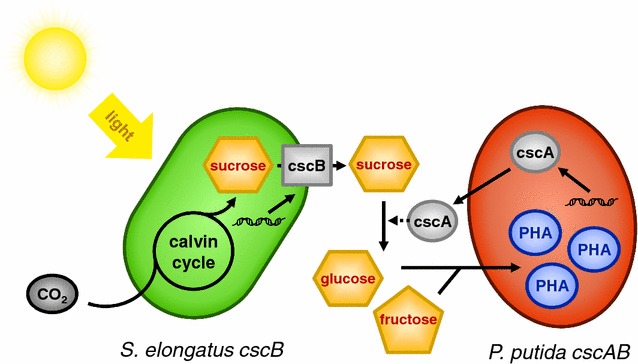
Concept of the synthetic co-culture of *S. elongatus cscB* and *P. putida cscAB* for the production of PHA from CO_2_ and light. CO_2_ is fixed via the Calvin cycle to make sucrose, which in turn is secreted into the surrounding medium by the activity of the heterologous sucrose permease CscB. CscA produced by *P. putida cscAB* leaks out of the cell, where it splits sucrose extracellularly [16]. The monomers (glucose and fructose) are metabolized by *P. putida cscAB,* and polyhydroxyalkanoates (PHA) are accumulated in the cytoplasm [15]

In this work, we aimed to produce biodegradable plastic from light and CO_2_, tackling two of the major challenges of modern times: global warming and pollution by plastics. Strategies to combat these problems are the fixation of CO_2_ to avoid its emission into the atmosphere and to find economically competitive processes for the production of plastic substitutes like PHA. Here, we present an approach in which the sucrose production of *S. elongatus cscB* is directly coupled to PHA accumulation by *P. putida cscAB* in a synthetic co-culture. Thus, CO_2_ and sunlight are converted into carbon neutral bioplastics (Fig. 1). *P. putida* is known for its innate stress resistance and robustness and is therefore an excellent candidate as a mixed culture partner. The strain *P. putida cscAB* is genetically modified to be able to metabolize sucrose [15, 16], and is the strain of choice in this co-culture. We present the steps undertaken towards a functional mixed culture and the first improvements made to considerably increase the PHA content of the cells (to about 150 mg/L at the end).

## Results and discussion

### Sucrose production of *S. elongatuscscB* in BG-11^+^ medium

**Fig. 2:**
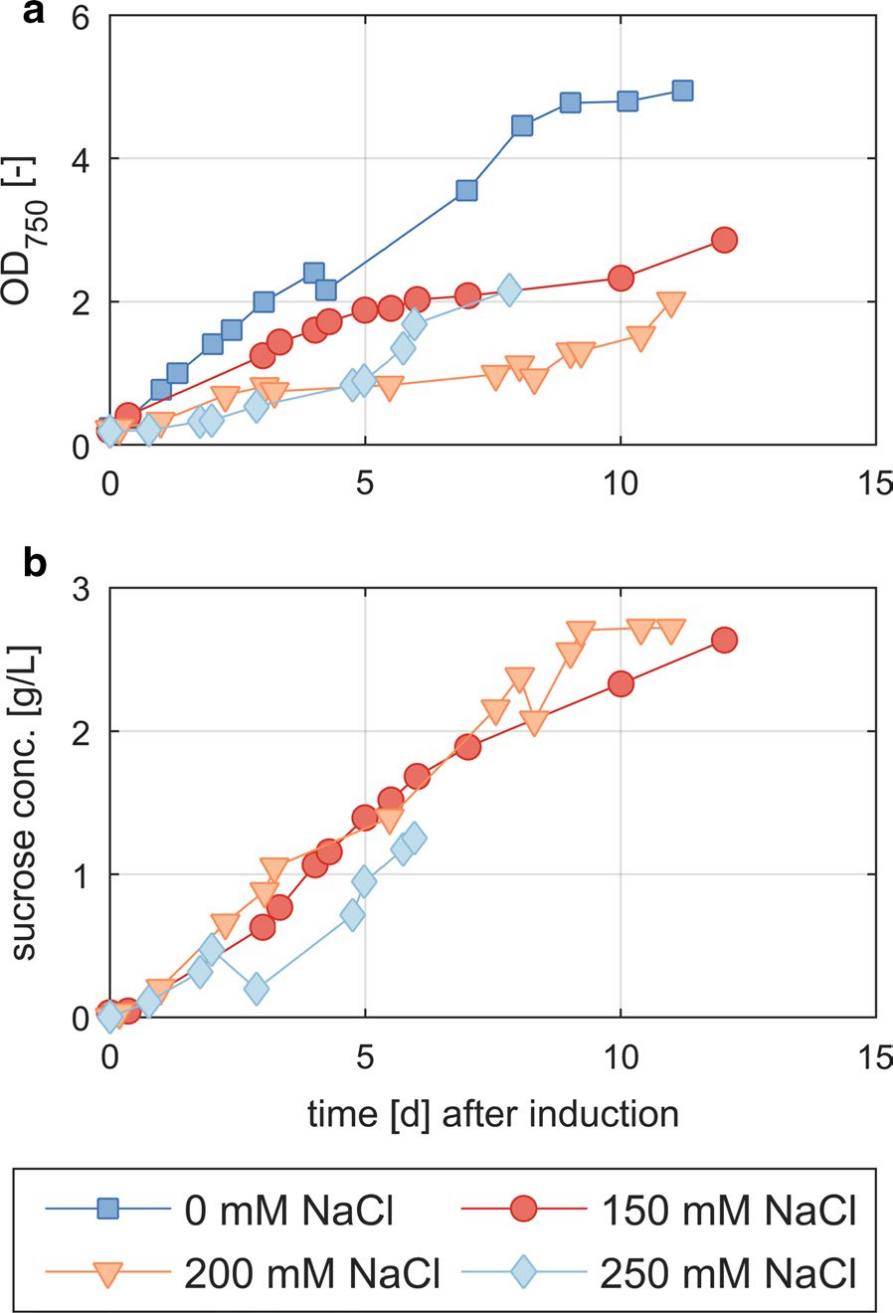
Growth and sucrose concentration from fermentations of *S. elongatus cscB* in a photobioreactor in BG-11^+^ in the presence of different salt concentrations. Cells were grown in BG11^+^ medium with the NaCl concentration indicated, and growth and sucrose secretion were monitored. CscB production was induced with 0.1 mM IPTG at an OD of 0.1–0.2

As *S. elongatus* accumulates sucrose as a compatible solute in response to the external salt concentration, we set out to identify the optimal NaCl concentration that would allow maximal sucrose excretion without severely inhibiting growth. Recently, it was shown that in normal BG-11 medium, *S. elongatus cscB* showed the highest sucrose production in the presence of 150 mM NaCl [6]. However, as the medium in this study was modified to meet the needs of both co-culture partners (see “Methods”) and the cultivation conditions were different, the influence of NaCl on sucrose production and growth had to be assessed again in the newly defined medium in a 1.8 L photobioreactor at controlled pH. Therefore, *S. elongatus cscB* was cultivated in BG-11^+^ medium in the absence or presence of NaCl in concentrations ranging from 150 to 250 mM. Growth of the cells and excretion of sucrose were monitored by measuring the optical density and determining the concentrations of sucrose in the supernatants by HPLC (Fig. 2). The growth of *S. elongatus cscB* is clearly reduced by an increase in the external salt concentration, ranging from a biomass production rate of 0.236 g CDW/L d without NaCl to 0.059 g CDW/L d in the presence of 250 mM NaCl during light-limited, linear growth (Table 1). The highest sucrose productivity, however, was observed with 150 mM NaCl, which is in accordance with what was reported by Ducat et al. for normal BG-11 medium [6]. We reached a production rate of 0.346 ± 0.014 g/(L day) and a maximal titer of 2.63 g/L after 12 days (Table 1). By comparing the amount of sucrose produced under salt stress to the biomass production without NaCl, the carbon flux in the cyanobacteria is mirrored: At 150 mM NaCl, the produced mass of sucrose per unit time is higher than the amount of biomass produced without NaCl, indicating that a large fraction of the primary production is directed towards the synthesis of the compatible solute sucrose.

**Table 1:** Maximal linear growth and sucrose production rates of *S. elongatus cscB* at different NaCl concentrations in BG-11^+^ medium

NaCl (mM)	Biomass production rate^a^ [g_CDW_/(L day)]	Sucrose production rate^a^ [g/(L day)]
0	0.236 ± 0.00400	n.d.
150	0.134 ± 0.004	0.346 ± 0.014
200	0.116 ± 0.008	0.282 ± 0.012
250	0.059 ± 0.005	0.20 ± 0.03

^a^Standard deviations are regression errors, not derived from replicates

### Growth of *P. putida cscAB* in modified BG-11 media

**Fig. 3:**
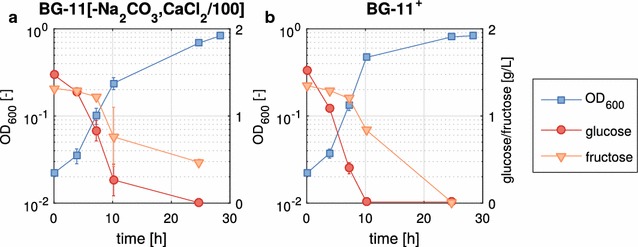
Growth of *P. putida cscAB* in modified cyanobacterial BG-11 medium supplemented with glucose, fructose (each 1.5 g/L), and 150 mM NaCl. Experiments were conducted in 250 mL unbaffled shake flasks with an effective volume of 25 mL. Temperature was set to 30 °C, and agitation rate to 220 rpm. Shown are the means and standard deviations calculated from three biological replicates

Next, we had to confirm that the metabolically engineered *P. putida cscAB* is able to grow in the modified BG-11 medium in the presence of 150 mM NaCl, the optimal salt concentration for sucrose production by *S. elongatus cscB*. Additionally, the influence of the changes in the medium composition had to be examined by comparing both BG-11 derived media. Therefore, *P. putida cscAB* was grown in BG-11[–NaCO_3_, CaCl_2_/100] and BG-11^+^, in the presence of 150 mM NaCl and a mixture of glucose and fructose (each 1.5 g/L) as the carbon source (Fig. 3). We chose the monomers of sucrose as the carbon source to assay solely the effect of the medium composition on the growth of *P. putida cscAB* and not the influence of the efficiency of sucrose splitting. The strain grew well in both media with a growth rate of 0.239 ± 0.001/h for BG-11[–NaCO_3_, CaCl_2_/100] and 0.305 ± 0.011/h for BG-11^+^. The slight increase in the growth rate in BG-11^+^ can most likely be attributed to the higher nutrient availability as the concentrations of potassium phosphate and magnesium sulfate were increased tenfold in this medium. This suggests that one or both of these nutrients are limiting factors in the original medium. The growth rate obtained in BG-11^+^ was in the same range as the one determined in M9 medium [16], which is the standard minimal medium for *P. putida*. Thus, only minor adjustments in medium composition were necessary to achieve comparable growth of *P. putida cscAB* in BG-11 as well. This reflects the broad metabolic versatility and robustness of this bacterium. Other bacteria and eukaryotic culture partners seem to be less suited for co-cultivation in a photosynthetic consortium as they have higher nutritional demands. Recently, Hays et al. reported co-cultivation of three heterotrophs with *S. elongatus cscB.* However, in their process, the medium was also supplemented with ammonium salts and buffer [9]. The same seems to be true for oleaginous yeast as described by Li et al. [17]. As outlined above, supplementation of the medium with additives was not necessary with *P. putida cscAB* as co-cultivation partner. This underlines the versatility and suitability of *P. putida* as a chassis for industrial biotechnology [18].

### Mixed culture of *S. elongatus cscB* and *P. putida cscAB*

**Fig. 4:**
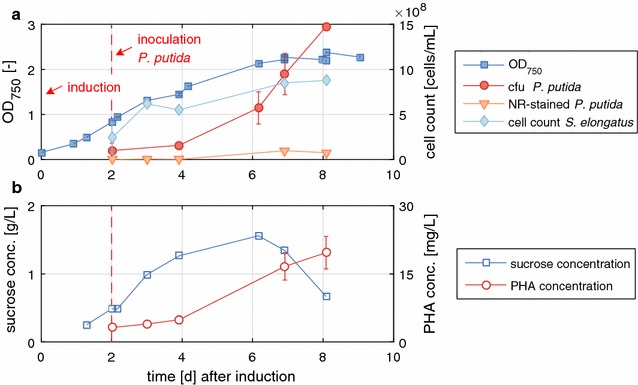
Co-cultivation of *S. elongatus cscB* and *P. putida cscAB* in the photobioreactor. Optical density, Nile red-stained cell count of *P. putida cscAB*, cell count of *S. elongates cscB* and colony forming units (CFU) determined from plating are depicted over time in **a**. *Arrows* indicate the induction of sucrose export by the addition of 0.1 mM IPTG and inoculation of *P. putida cscAB*. Sucrose and PHA concentrations are plotted in **b**. Uncertainties in PHA concentrations were estimated from the propagation of errors of the PHA standards

Having two strains with complementary functions, one producing sucrose from CO_2_, the other consuming sucrose, and a common medium allowing the growth of both, we set out to grow them simultaneously in the same cultivation vessel. Each strain represents a functional bio-module, which is linked to the other by sucrose transfer. To start the synthetic mixed culture, first *S. elongatus cscB* was inoculated in a photobioreactor in BG-11^+^ in the presence of 150 mM NaCl to promote sucrose accumulation right from the beginning. The heterotrophic organism was inoculated at least 1 day after induction of sucrose export with 0.1 mM IPTG to ensure that sucrose was readily available for *P. putida cscAB*. The total OD of the culture was monitored, i.e., the sum of *S. elongatus cscB* and *P. putida cscAB* cells, as well as the concentration of sucrose in the culture supernatant (Fig. 4). To have an approximation of the contribution of each strain to the overall OD, the colony forming units (CFUs) of each strain, and the cell counts of Nile red-stained *P. putida cscAB* were determined. Nile red predominantly stains lipophilic residues like PHAs [19] therefore, the number of cells reported represents only the proportion of *P. putida cscAB* cells that accumulated PHA in their cytoplasm. The overall OD of the mixed culture increased over a period of 7 days reaching a plateau of about OD = 2.3 at the end of the process (Fig. 4a). The growth behavior of *S. elongatus cscB* was similar to the process in pure culture at 150 mM NaCl (compare Figs. 2, 4a), hence there seems to be no severe negative effect from the presence of the commensal *P. putida cscAB*. Recently, co-cultivation of *S. elongatus cscB* with other organisms even showed a positive effect on the growth of the cyanobacterium [9]. Sucrose accumulated steadily up to 1.5 g/L in the culture supernatant until day 6. This is when *P. putida cscAB* reached a critical mass, and when sucrose started to be metabolized more rapidly than it was built. As the cell counts of *P. putida cscAB* increased, sucrose concentrations decreased, but no extracellular glucose or fructose accumulation was detected. Transient accumulation of the sugar monomers was observed in earlier studies, when *P. putida cscAB* was grown in M9 medium with sucrose as the sole carbon source [16]. It was attributed to the extracellular cleavage of sucrose by invertase CscA that was leaking out of the cells. Therefore, sucrose splitting seems to be the limiting factor for the growth of *P. putida cscAB*, as no sugar monomers were accumulated, and as with sucrose as C-source the growth rate was markedly reduced compared to a mixture of glucose and fructose in BG-11^+^ (compare to Fig. 3). Along the same line, pure cultures of *P. putida cscAB* in BG-11^+^ medium with sucrose as the sole carbon source showed inconsistent and very slow growth (data not shown). Thus, when sucrose is used as carbon source, the medium composition clearly has an influence on the growth of *P. putida cscAB*. However, the controlled process parameter in the photobioreactor and maybe the presence of *S. elongatus cscB* seem to have a stabilizing effect on *P. putida cscAB*, so that reliable growth in the co-culture was achieved. Nevertheless, we assume that the growth of *P. putida cscAB* can be enhanced when sucrose is metabolized more efficiently. The sugar was detectable in the supernatant during the whole co-cultivation period, thus it was not consumed completely. This shortcoming will be tackled by the development of new sucrose splitting variants of *P. putida* with a higher splitting rate, which can be obtained for instance by active secretion of the invertase or screening for other sucrose permeases.

We also analyzed the PHA content of the cells, as *P. putida* accumulates these polymers to a small extent even under non-standard PHA accumulating conditions [20, 21]. *P. putida cscAB* produced PHA in the mixed culture at an approximate maximal production rate of 3.3 mg/(L day) and a maximal titer of 19.7 mg/L was reached 6 days after inoculation with *P. putida cscAB* (Fig. 4b). It can be excluded that *S. elongatus cscB* is responsible for PHA formation since it lacks the corresponding genes for PHA synthesis [22]. Moreover, the distribution pattern of 3-hydroxyalkanoic acids is typical for *P. putida* (Table 2), with 3-hydroxydecanoic acid being the most abundant monomer [23]. Apparently, PHA was accumulated in only a small fraction of the cells, which can be deduced when comparing the CFU/mL to the cell counts of Nile red-stained cells. On day 8, when the highest PHA titer was detected, only a very small fraction of the *P. putida cscAB* cells were stained with Nile red, i.e., accumulated PHA in their cytoplasm. To provide absolute numbers, further experiments, including counting cells with PHA granules under a microscope and comparing the numbers to counts gained by flow cytometry, are necessary to determine the efficiency of the staining method with Nile red. The flow cytograms, however, do provide qualitative information about the specific fluorescence and thus the PHA content of the stained cells, clearly showing the presence of PHA (see Additional file 1: Figure S2).

**Table 2:** Distribution of chain-lengths per mass fraction in PHA produced by *P. putida cscAB* in the mixed culture at maximal PHA concentration during the process

Chain-length (carbon number)	6	8	10	12	12:1
Mass fraction (%)	4.2	25.2	58.4	4.4	7.8

This way, the general process parameters for co-cultivation of *S. elongatus cscB* and *P. putida cscAB* for the production of PHAs from CO_2_ and light were set and the feasibility of the process was confirmed. Without adding any other carbon source than CO_2_ to the process, PHAs were produced by *P. putida*, however, yield and production rate were low.

### Polyhydroxyalkanoate production in the co-culture under nitrogen-limiting conditions

One way to improve the PHA accumulation is to alter the C/N ratio of the growth conditions. Although not strictly necessary for PHA accumulation, nitrogen limitation in general increases the PHA content of the cells [24]. Therefore, we conducted the mixed culture experiment under nitrogen-limiting conditions. An initial batch phase was performed in which *S. elongatus cscB* was grown with a starting nitrate concentration of 48.0 mg/L and a nitrate feed of 9.2 ± 0.4 mg/day. Four days after induction of sucrose export with 0.1 mM IPTG and upon inoculation of the co-culture partner *P. putida cscAB*, the HNO_3_-feed was increased to a rate of 46 ± 2 mg/day (NO_3_^−^). The nitrogen concentration was chosen so that it was just below the threshold that allows unlimited growth of *S. elongatus cscB*. Ideally, this should keep the steady-state concentration of nitrogen low enough to promote PHA formation by *P. putida cscAB*.

**Fig. 5:**
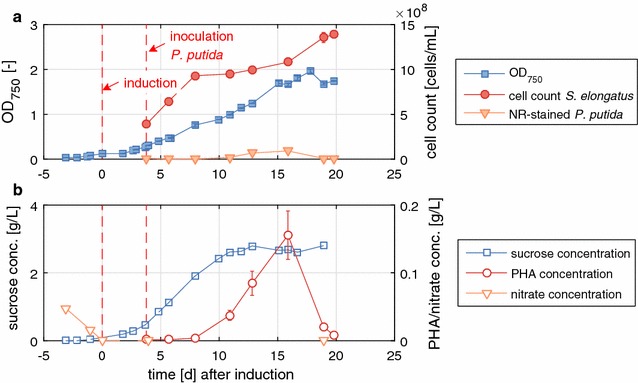
Nitrate-limited co-cultivation of *S. elongatus cscB* and *P. putida cscAB*. Optical density, Nile red-stained cell count of *P. putida cscAB* and cell count of *S. elongatus cscB* are depicted over time in **a**. Sucrose, PHA and nitrate concentrations are plotted in **b**. Uncertainties in PHA concentrations were estimated from the propagation of errors of the PHA standards. Nitrate was fed at a constant rate of 46 ± 2 mg/day after inoculation with *P. putida cscAB*

The results of this cultivation are illustrated in Fig. 5. After an initial period of 4 days, nitrate was no longer detectable in the photobioreactor, and is therefore, considered completely consumed by *S. elongatus cscB*. This was the time point of induction of *cscB* expression and is defined as day 0. The optical density started to rise, i.e., *S. elongatus cscB* started to grow, and on day 4, *P. putida cscAB* was inoculated. As expected, *S. elongatus cscB* grew linearly (dOD/dt = 0.105 ± 0.005/day during days 4–8, Fig. 5), although at a lower rate than under unlimited conditions (dOD/dt = 0.38 ± 0.03/day during days 1–4, Fig. 4). Sucrose was secreted constantly, reaching a maximal production rate of 0.316 g/(L day) and a maximal titer of 2.8 g/L 10 days after induction. The production rate reached was only marginally lower than the one obtained under non-limiting conditions (0.346 g/(L day), compared in Table 1). *P. putida cscAB* was apparently very stressed, which was manifested in non-reliable growth on LB agar plates when determining the CFUs (data not shown). Therefore, we could not use the cell count data from plating as an approximation of cells present in the co-culture. This behavior may be a result of the cumulative stress of a poor nitrogen source, nutrient limitation, low carbon availability because of low sucrose splitting rate, and possibly salt stress due to the presence of 150 mM NaCl in the medium. However, counting the Nile red-stained cells of *P. putida cscAB* by flow cytometry gave a qualitative estimate, and cell counts showed a steady course over time (Fig. 5a), which correlated nicely with the PHA concentration measured (Fig. 5b). The actual number of *P. putida cscAB* cells might be higher, assuming that only a fraction of the culture accumulates the polymer or are stained by the staining method. PHA accumulating cells as well as the PHA concentration increased until day 16, and then both decreased again. One possible explanation for the decrease might be cessation of sucrose metabolism and the consumption of intracellular reserves of PHAs, since it is possible that the actual number of *P. putida cscAB* cells continued to increase. PHA production reached a maximal production rate of 23.8 ± 6 mg/(L day) and a maximal titer of 156 ± 40 mg/L after 16 days. Thus, by applying nitrate limitation, we could increase the production rate about 7.3-fold.

To place these numbers in context: The achieved PHA level in the nitrogen-limited process exceeded the values reported for the mixed cultures of *S. elongatus cscB* with *E. coli* or *A. vinelandii* about a 150-fold [9, 14]. There are also efforts to produce PHB directly with recombinant cyanobacteria [25, 26], in which a high-cell dry weight fraction of PHB is already reached. However, there is no information about productivity available, and hence it cannot be determined how those strains compare to the process presented in this work.

A general challenge when working with phototrophic organisms is to reach the high-cell densities needed for efficient downstream processing. One problem is self-shading, which in the approach presented here could be solved by a compartmentalization of *S. elongatus cscB* and *P. putida cscAB.* In the process presented here, a major fraction of sucrose was left untouched by *P. putida cscAB*, which leaves room for the production of even more PHA. This might be facilitated by the construction of *P. putida* strains that split sucrose more efficiently. Furthermore, PHA production by *P. putida* can also be increased by metabolic engineering to direct more carbon flux to PHA formation [27] and thus reach higher PHA weight fractions of the cell dry weight, or by increasing the fraction of the population that actually accumulates PHAs. These attempts are currently under investigation in our laboratory.

## Conclusion

In the work presented here, a mixed culture of *S. elongatus cscB* and *P. putida cscAB* was established in a lab-scale photobioreactor for the production of PHAs, a green plastic substitute. The concept of coupling a photosynthetic organism to a heterotrophic bacterium with the aim to produce an industrially relevant compound was successful and makes room for the implementation of a wide repertoire of bioproducts that can be produced by *P. putida* [18, 28]. Only slight adjustments in the cyanobacterial growth medium composition were necessary to promote growth and PHA production by *P. putida cscAB*, showing the universality and robustness of this organism. When evaluating the productivity and yields of the co-culture, it has to be kept in mind that neither the process nor the organisms were optimized yet, which leaves great potential for optimization: One possible target is to increase the sucrose production by *S. elongatus cscB* through metabolic engineering, optimizing light influx or the use of higher photon flux densities. Indeed, under natural conditions at well-suited sites, photon flux densities are higher than the ones used in this work [29], which could result in an increased photosynthetic carbon fixation rate. Other potential targets include the improvement of sucrose consumption by *P. putida cscAB* or the more efficient channeling of the carbon flux towards PHA formation to achieve a higher weight fraction of PHA relative to the cellular dry weight. Here, we reached a maximal concentration of ~150 mg/L PHA, which is quite low, compared to pure cultures. However, this was achieved with a small number of cells compared to the cell densities reached in pure cultures, which are normally cultivated in fed-batch, high-cell density processes [23]. Nevertheless, it is crucial for efficient downstream processing to obtain a high concentration of PHA. One way to accomplish higher cell densities in coupled cultures is to separate the autotrophic and heterotrophic processes into different compartments [30] or to extend the process duration. In a possible future scenario, sunlight will be collected by a large area of photobioreactors and product formation by *P. putida* will be mainly limited by the sucrose production of *S. elongatus cscB*. For efficient downstream processing, *P. putida* could be either upconcentrated, e.g., by cultivation in a membrane bioreactor or hydrogel as suggested by Smith and Francis [14], or it could be auto-aggregated as described for other bacteria [31] and collected by decanting. Consequently, titers and production rates here cannot, and maybe do not have to, be compared to those of contemporary studies on PHA production in pure cultures. Future experiments with set-ups closer to a possible application scenario will elucidate the feasibility of this technology. In the future, the concept of the modular co-culture might also serve as a platform process with *S. elongatus cscB* fixing CO_2_ and converting it to sucrose and *P. putida cscAB* serving as chassis for the implementation of synthetic pathways. This way, the product spectrum could be amplified tremendously.

In the long run, the applicability of commodities produced by microalgae will stand or fall depending on many factors: the development of cheaper and more efficient photobioreactors, the long-term dominance of cultures versus contaminations, the genetic streamlining of organisms, energy costs, the development of power-to-chemical processes and the price of oil, just to name a few. Whichever renewable technology will be feasible in the future, all research efforts in this area are important, as one fact is evident: availability of fossil resources will come to an end.

## Methods

### Bacterial strains and batch cultivation

Two organisms were used in the mixed culture: The autotrophic host, *S. elongatus cscB,* was kindly provided by Pamela Silver [6]. The heterotrophic commensal was *P. putida* EM178 *att*::miniTn7(eYFP) PP_3398::*cscAB*, a derivative of the prophage-free KT2440 strain EM178 [16]. For reasons of simplicity the strain will be called *P. putida cscAB.* Pre-cultures of *S. elongatus cscB* were grown in 100 mL shaking flasks with 40 mL of BG-11[–NaCO_3_, CaCl_2_/100] medium at 100 rpm, at 30 °C and a photon flux density of approximately 10–26 µmol/(m^2^ s). A 30 W tubular fluorescent lamp was used for lighting, and air was the only source of CO_2_. Experiments with *P. putida* alone were conducted in 250 mL flasks with 25 mL of BG-11[–NaCO_3_, CaCl_2_/100] or BG-11^+^ at 30 °C under shaking. Pre-cultures for *P. putida* experiments were grown in LB-medium overnight and washed once in the cultivation medium of the main culture prior to inoculation.

### Media

As a first step towards a functional co-culture between S*. elongatus cscB* and *P. putida cscAB*, a common growth medium for both organisms had to be defined, using original BG-11 (ATCC Medium 616) medium for blue-green algae [32] as starting point. The pH was shifted towards neutral pH, by omitting sodium carbonate, and CaCl_2_ was reduced 100-fold to 3.4 µM, as it interfered with the growth of *P. putida* (see Additional file 1: Figure S1). This medium is referred to as BG-11[–NaCO_3_, CaCl_2_/100]. For its preparation four stock solutions were made, filter sterilized and stored at −20 °C in appropriate aliquots: solution 1 [100×]: 150 g/L NaNO_3_, 3 g/L K_2_HPO_4_; solution 2 [1000×]: 75 g/L MgSO_4_·7H_2_O, 5 g/L citric acid, 6 g/L iron–ammonium citrate; 1.1 g/L disodium ethylenediaminetetraacetate·2H_2_O; solution 3 [1000×]: 0.36 g/L CaCl_2_; trace element solution A5 [1000×]: 2.86 g/L H_3_BO_3_, 1.81 g/L MnCl_2_·4H_2_O, 0.222 g/L ZnSO_4_·7H_2_O, 0.39 g/L NaMoO_4_·2H_2_O, 0.079 g/L CuSO_4_·5H_2_O, 49.4 mg/L Co(NO_3_)_2_·6H_2_O. The medium was prepared by adding each stock solution to autoclaved deionized water to reach 1× concentration (10 mL/L of solution 1, and 1 mL/L of solutions 2–4, respectively).

Additionally, based on this, a second medium was designed by increasing the phosphate and sulfate concentrations tenfold to exclude limitations that might occur when adding an additional microbe to the culture.

For this purpose, the components K_2_HPO4 and MgSO_4_·7H_2_O were increased 10 times. They were added as separate, sterile stock solutions (300 g/L K_2_HPO_4_, 246.5 g/L MgSO_4_·7H_2_O). This enriched medium is designated herein BG-11^+^.

### Cultivation in a photobioreactor

Liter-scale cultivations of *S. elongatus cscB* and mixed cultures were performed in a Labfors 5 Lux flat panel airlift photobioreactor (Infors AG, Switzerland) at a photon flux density of approximately 240 µmol/(m^2^ s). The pH was controlled at 7.5 with 1 mol/L HNO_3_ and an airflow of 2 L/min, enriched with 2% CO_2_, was used as a carbon supply for autotrophic growth. The reactor was filled with 1.8 L of water containing the desired NaCl concentration, and autoclaved, and the medium ingredients were added sterilely through a septum from stock solutions. Twenty mL of a stationary *S. elongatus cscB* culture were used as inoculum and cells were grown until an optical density (OD_750_) of 0.1–0.2 (equals a CDW of about 0.04–0.08 g/L) was reached; then 0.1 mmol/L IPTG was added to induce expression of *cscB* and thus sucrose export. For mixed culture cultivation, an over-night culture of *P. putida cscAB* grown in BG-11^+^ medium supplemented with 3 g/L glucose H_2_O was then washed once with 5 mL reactor medium and added to the reactor.

In the case of the nitrate-limited process, the initial nitrate concentration was reduced to 50 mg/L NO_3_^−^. Upon inoculation with *S. elongatus cscB* a constant nitrate feed of 9.2 ± 0.5 mg/day was implemented by pumping a 0.03 mol/L HNO_3_ solution into the reactor vessel. After inoculation with *P. putida cscAB,* the nitrate feed was increased to 46 ± 2 mg/day.

Samples were taken to measure the optical density, and culture supernatants were frozen and stored for subsequent HPLC analysis. At least every third day cells were diluted and the composition of mixed cultures was assessed by flow cytometry. The pellets of 5 mL of every sample were frozen at −80 °C for GC analysis. Growth of heterotrophic populations was additionally followed via plating in suitable dilutions on LB-medium agar plates.

### Optical density, cell counting and determination of cell dry weight

The optical density of 200 µL culture was measured with an Infinity^®^ microplate reader (Tecan, Austria) at wavelengths of 600 (*P. putida cscAB* cultivation) and 750 nm (*S. elongatus cscB* and mixed cultures). Sucrose, glucose, and fructose concentrations were determined by high performance liquid chromatography (HPLC) using an Agilent machine (Agilent 1100 series). Sugars were separated via a Shodex SH1011 column and a mobile phase of 0.5 mmol/L H_2_SO_4_ at a flow rate of 0.5 mL/min and a temperature of 30 °C. For flow cytometry, cultures were diluted to reach a cell count of about 800–1400 at a flow rate of 1–5 µL/s and injected into a CyFlow^®^ instrument (Sysmex Partec GmbH, Germany) equipped with a laser (488 nm excitation wavelength). Fluorescence was measured at 536, 590, and 630 nm emission wavelengths. One mL of PHA-containing cells was centrifuged, stained with 10 µL Nile red solution (1 g/L in DMSO) and incubated for 5 min prior to dilution and followed by PHA-mediated Nile red fluorescence at 590 nm emission wavelength.

For cell dry weight measurement, an appropriate volume of cells was centrifuged at 8000×*g* for 10 min. The pellet was resuspended in phosphate buffered saline (PBS) and centrifuged again in a pre-dried and weighted 1.5 mL centrifuge tube. The supernatant was carefully discarded as completely as possible and the tube was dried at 60 °C for at least 3 days until the weight remained constant. The weight difference represented the dry weight. A correlation between optical density and cell dry weight was made for both organisms (data not shown) and cell dry weights of the other experiments were estimated from this correlation.

### PHA determination

PHA content and composition were determined by gas chromatography (GC). A modified version of the propanylation protocols by Riis and Mai [33] and Furrer et al. [34] was applied. Samples were centrifuged at 17,000×*g* for 5 min and pellets were frozen and stored at −80 °C. To remove residual water, samples were freeze-dried for at least 3 days. The samples were dissolved in 2 mL of chloroform in an Ace^®^ overpressure glass tube, and subsequently 1 mg of Poly-3-hydroxybutanoate (Sigma-Aldrich) and 0.2 mg of 3-methylbenzoic acid (Sigma-Aldrich) were added as internal standards. Two mL of an 80% (v/v) solution of 1-propanol and 37% HCl were pipetted into the mixture and the tube was sealed tightly. The bottom third of the tube was placed in an oil bath at 80 °C and mixed using a magnetic stirrer. After 16–24 h the tube was cooled to room temperature and 4 mL of bidistilled water were added. After shaking the tube vigorously, the tube was left at room temperature until the phases separated. The upper, aqueous phase was removed carefully, and the remaining liquid was dried with Na_2_SO_4_ and neutralized with Na_2_CO_3_. The remaining organic layer was transferred to a GC vial and injected into the GC machine (injection volume 1 µL). The samples were separated with a fused silica Stabilwax^®^ column (Restek AG, Fuldabrueck, Germany) and measured with a flame ionization detector (detector temperature 245 °C). A temperature of 240 °C was set for the split/splitless injector (split ratio 1:10). Hydrogen gas was used as carrier gas at a flow rate of 3 mL/min. The different 3-hydroxyalkanoic acid esters were separated by applying a temperature gradient, starting at 80 °C (1 min), which then increased 5 °C every minute, stopping at 240 °C (hold time 5 min).

3-hydroxydecanoic acid (Sigma-Aldrich) and PHB were used as external standards. The response factors of the remaining 3-hydroxyalkanoic and 3-hydroxyalkenoic acids were inter- or extrapolated linearly from the two standards, according to Tan et al. [35].

### Measurement of nitrate concentration

The nitrate/nitrite concentration of culture supernatants was determined using a colorimetric assay (Nitrite/Nitrate colorimetric method, Roche Diagnostics GmbH, Penzberg, Germany) in a microplate reader based on the enzymatic reduction of nitrate to nitrite. Nitrite reacts with a combination of dyes to form a diazo dye that can be measured at a wavelength of 540 nm and correlates linearly with the original nitrate/nitrite concentration.

## Additional file

**Additional file 1: Figure S1.** Reduction of the CaCl_2_ concentration promotes growth of *P. putida* EM178 in BG-11 [–NaCO_3_] medium. Growth of *P. putida* EM178 with different concentrations of CaCl_2_. Experiments were performed in 100 mL, unbaffled shake flasks filled with 10 mL of medium at 30 °C and an agitation rate of 220 rpm. Note that at 3.4 μM CaCl_2_, the concentration chosen for BG11^+^, no limitation in growth of *P. putida* EM178 is observed. **Figure S2.** Exemplary flow cytogram of Nile red-stained cells during nitrate-limited mixed culture of *P. putida cscAB* and *S. elongatus cscB* at the maximal concentration of PHA. The cells marked in the red circle only appeared upon staining with Nile red and are not found in the unstained control (data not shown). Cells below are unstained cells of both strains and undefined background.

## Abbreviations

PHA: polyhydroxyalkanoate
PHB: polyhydroxybuturate
CFU: colony forming units
GC: gas chromatopraphy
PBS: phosphate buffered saline
HPLC: high performance liquid chromatography
CDW: cellular dry weight
DMSO: dimethyl sulfoxide
