# Supplementary material for: Photoautotrophic production of polyhydroxyalkanoates in a synthetic mixed culture of Synechococcus elongatus cscB and Pseudomonas putida cscAB
Source: Biotechnol Biofuels. 2017 Jul 19;10:190. doi: 10.1186/s13068-017-0875-0 (PMC5517840; doi:10.1186/s13068-017-0875-0)
Supplement: Supplementary file 1 — Additional file 1: Figure S1. Reduction of the CaCl2 concentration promotes growth of P. putida EM178 in BG-11 [–NaCO3] medium. Growth of P. putida EM178 with different concentrations of CaCl2. Experiments were performed in 100 mL, unbaffled shake flasks filled with 10 mL of medium at 30 °C and an agitation rate of 220 rpm. Note that at 3.4 μM CaCl2, the concentration chosen for BG11+, no limitation in growth of P. putida EM178 is observed. Figure S2. Exemplary flow cytogram of Nile red-stained cells during nitrate-limited mixed culture of P. putida cscAB and S. elongatus cscB at the maximal concentration of PHA. The cells marked in the red circle only appeared upon staining with Nile red and are not found in the unstained control (data not shown). Cells below are unstained cells of both strains and undefined background. [file 13068_2017_875_MOESM1_ESM.pdf]

Additional file 1

Photoautotrophic Production of Polyhydroxyalkanoates in a Synthetic Mixed Culture of *Synechococcus elongatus cscB* and *Pseudomonas putida cscAB*

Hannes Löwe, Karina Hobmeier, Manuel Moos, Andreas Kremling, and Katharina Pflüger-Grau *

Fachgebiet für Systembiotechnologie, Technische Universität München, 85748 Garching (Germany)

---

* Correspondence to: Katharina Pflüger-Grau
Fachgebiet für Systembiotechnologie
Technische Universität München
Boltzmannstr. 15
85748 Garching
Tel.: +49 89 289 15765; Fax.: +49 89 289 15766
Email: k.pflueger-grau@tum.de

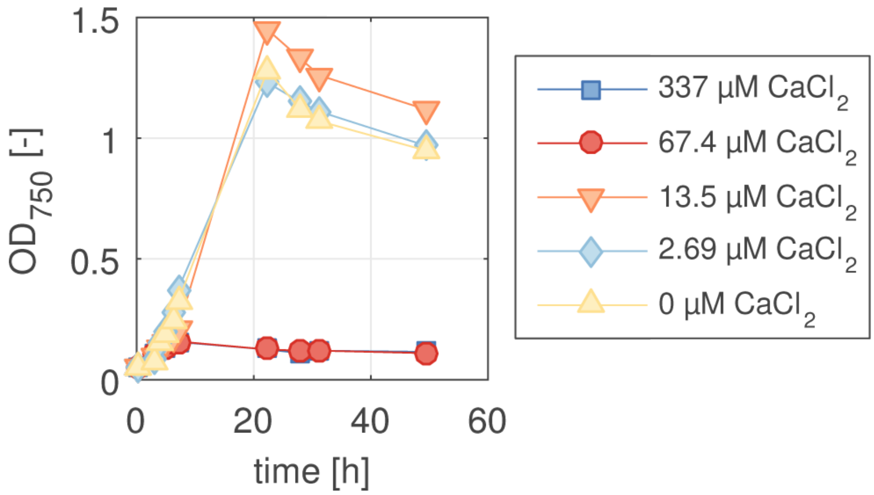

Figure S1: Reduction of the $CaCl_2$ concentration promotes growth of *P. putida* EM178 in BG-11 [-$NaCO_3$] medium. Growth of *P. putida* EM178 with different concentrations of $CaCl_2$. Experiments were performed in 100 mL, unbaffled shake flasks filled with 10 mL of medium at 30°C and an agitation rate of 220 rpm. Note that at 3.4 µM $CaCl_2$, the concentration chosen for $BG11^+$, no limitation in growth of *P. putida* EM178 is observed.

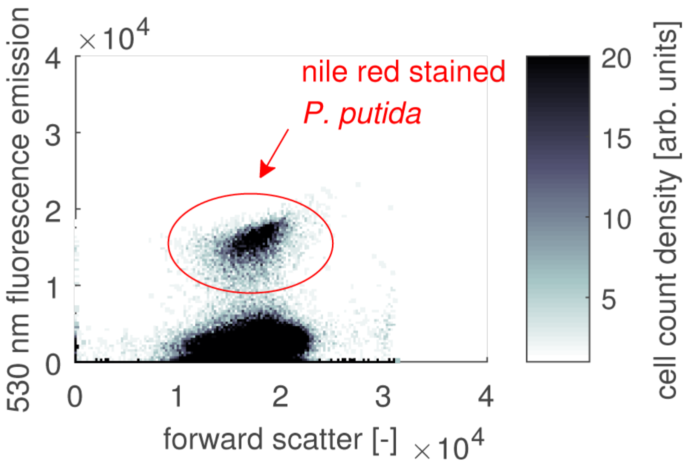

Figure S2: Exemplary flow cytogram of Nile red stained cells during nitrate-limited mixed culture of *P. putida cscAB* and *S. elongatus cscB* at the maximal concentration of PHA. The cells marked in the red circle only appeared upon staining with Nile red and are not found in the unstained control (data not shown). Cells below are unstained cells of both strains and undefined background.
